# 2-Amino-5-methyl-6-methyl­sulfanyl-4-phenyl­benzene-1,3-dicarbonitrile

**DOI:** 10.1107/S1600536811055243

**Published:** 2012-01-18

**Authors:** Xuan Liu, Jianhong Tang, Yunqiao Huang, Huawei Zhang, Jiarong Li

**Affiliations:** a School of Chemical Engineering and Environment, Beijing Institute of Technology, Beijing 100081, People’s Republic of China; b College of Chemical Engineering, Huaqiao University, Xiamen Fujian 362021, People’s Republic of China

## Abstract

The dihedral angle between the planes of the two aromatic rings of the title compound, C_16_H_13_N_3_S, is 56.7 (3)°. The crystal packing is stabilized by inter­molecular N—H⋯N hydrogen bonds, which link the mol­ecules into chains along [11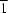].

## Related literature

For medicinal and biological properities of aromatic *o*-amino dinitrile derivatives, see Singh *et al.* (2009 ▶); Goel & Singh (2005 ▶); Pratap & Ramb (2008 ▶). For a related structure, see Singh *et al.* (2006 ▶).
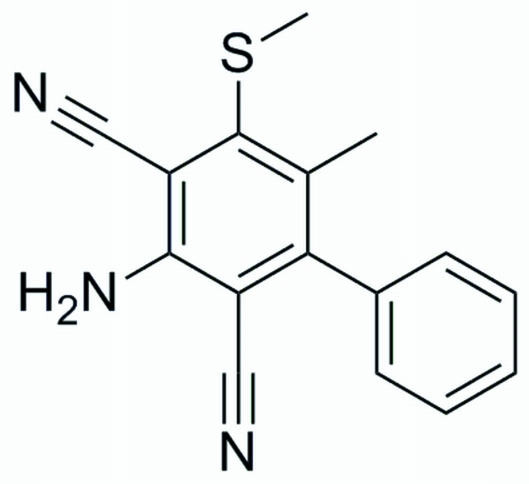


## Experimental

### 

#### Crystal data

C_16_H_13_N_3_S
*M* *_r_* = 279.35
Triclinic, 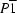

*a* = 8.959 (2) Å
*b* = 9.123 (2) Å
*c* = 10.1240 (19) Å
α = 65.843 (7)°
β = 68.362 (8)°
γ = 88.754 (10)°
*V* = 693.6 (3) Å^3^
*Z* = 2
Mo *K*α radiation
μ = 0.23 mm^−1^
*T* = 153 K
0.50 × 0.18 × 0.07 mm

#### Data collection

Rigaku AFC10/Saturn724+ diffractometer
Absorption correction: multi-scan (*CrystalClear*; Rigaku, 2008 ▶) *T*_min_ = 0.896, *T*_max_ = 0.985
7468 measured reflections
3601 independent reflections
2825 reflections with *I* > 2σ(*I*)
*R*_int_ = 0.023

#### Refinement

*R*[*F* ^2^ > 2σ(*F* ^2^)] = 0.037
*wR*(*F* ^2^) = 0.092
*S* = 1.00
3601 reflections
191 parameters
H atoms treated by a mixture of independent and constrained refinement
Δρ_max_ = 0.42 e Å^−3^
Δρ_min_ = −0.22 e Å^−3^

### 

Data collection: *CrystalClear* (Rigaku, 2008 ▶); cell refinement: *CrystalClear*; data reduction: *CrystalClear*; program(s) used to solve structure: *SHELXS97* (Sheldrick, 2008 ▶); program(s) used to refine structure: *SHELXL97* (Sheldrick, 2008 ▶); molecular graphics: *Mercury* (Macrae *et al.*, 2006 ▶); software used to prepare material for publication: *publCIF* (Westrip, 2010 ▶).

## Supplementary Material

Crystal structure: contains datablock(s) I, global. DOI: 10.1107/S1600536811055243/fy2026sup1.cif

Structure factors: contains datablock(s) I. DOI: 10.1107/S1600536811055243/fy2026Isup2.hkl

Supplementary material file. DOI: 10.1107/S1600536811055243/fy2026Isup3.cml

Additional supplementary materials:  crystallographic information; 3D view; checkCIF report

## Figures and Tables

**Table 1:** Hydrogen-bond geometry (Å, °)

*D*—H⋯*A*	*D*—H	H⋯*A*	*D*⋯*A*	*D*—H⋯*A*
N2—H2*B*⋯N1^i^	0.849 (17)	2.305 (17)	3.1360 (17)	166.2 (14)
N2—H2*A*⋯N3^ii^	0.872 (17)	2.253 (18)	3.0993 (17)	163.4 (15)

Symmetry codes: (i) 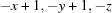; (ii) 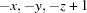.

**Fig. 1.**
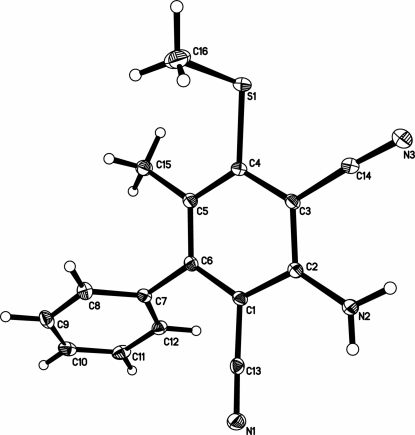
ORTEP diagram of (I) with ellipsoids drawn at the 50% probability level.

**Fig. 2.**
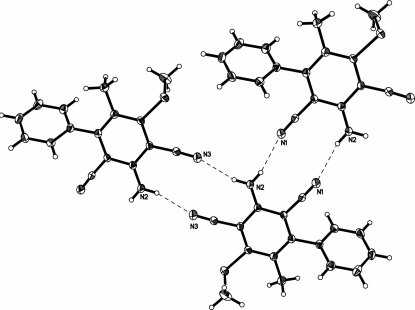
Hydrogen bonds in the crystal structure of (I).

## supplementary crystallographic information

### Comment

The title compound (I) was synthesized directly from the reation of 6-phenyl-5-methyl-4-methylsulfanyl-3-nitrile-2*H*-pyran-2-one and malononitrile. Its single-ctystal structure analysis was undertaken to comfirm its molecular structure and to determine the correlation of structural features with medical activity.

The molecular structure of (I) is shown in Fig. 1. The strong N—H···N intermolecular hydrogen-bonds are shown in Fig. 2. These hydrogen-bonds link the molecules into infinite chains along [1 1 -1].

### Experimental

The synthesis was based on the method of Singh *et al.* (2006). A mixture of 6-phenyl-5-methyl-4-methylsulfanyl-3-nitrile-2*H*-pyran-2-one (1 mmol), malononitrile (1.2 mmol), and powdered KOH (1.2 mmol) in dry DMF (5 ml) was stirred at room temperature for 5 h. At the end, the reaction mixture was poured into ice water with vigorous stirring for 4 h,and then filtered to give the title compound. The product was recrystallizated from ethanol to give yellow crystalline powder (m.p. 501–503 K).

50 mg of the product was dissolved in a mixed solvent (ethanol:petroleum ether 1:3) and was kept at room temperature for 4 days to give pale yellow single crystals.

Spectral data: IR (KBr): 3393, 3344, 2217, 1656, 1544, 1432, 1284, 836, 770, 698 cm^-1^; ^1H^-NMR(DMSO, p.p.m.): 1.47 (3*H*, s, CH_3_), 2.57 (3*H*, s, SCH_3_), 7.50–7.61 (5*H*, m, ArH), 8.29 (2*H*, s, NH_2_); ESI-MS m/*z*: [*M*+H]^+^ 280.1; C_16_H_13_N_3_S: calcd. C 68.79, H 4.69, N 15.04; found C 68.68, H 4.62, N 15.27.

### Refinement

Carbon-bound H atoms were included in the riding model approximation with C—H distances of 0.95–0.98 Å, and with *U*_iso_(H) = 1.2 *U*_eq_(C) or 1.5 *U*_eq_(*C*) for methyl groups. H atoms that are bonded to N2 were located by the difference Fourier method and were refined independently with isotropic displacement parameters.

### Crystal data

**Table:**

C_16_H_13_N_3_S	*F*(000) = 292
*M**_r_* = 279.35	*D*_x_ = 1.338 Mg m^−^^3^
Triclinic, *P*1	Melting point: 502 K
*a* = 8.959 (2) Å	Mo *K*α radiation, λ = 0.71073 Å
*b* = 9.123 (2) Å	Cell parameters from 2338 reflections
*c* = 10.1240 (19) Å	θ = 2.4–29.1°
α = 65.843 (7)°	µ = 0.23 mm^−^^1^
β = 68.362 (8)°	*T* = 153 K
γ = 88.754 (10)°	Chunk, colourless
*V* = 693.6 (3) Å^3^	0.50 × 0.18 × 0.07 mm
*Z* = 2	

### Data collection

**Table:**

Rigaku AFC10/Saturn724+ diffractometer	3601 independent reflections
Radiation source: rotating anode	2825 reflections with *I* > 2σ(*I*)
graphite	*R*_int_ = 0.023
Detector resolution: 28.5714 pixels mm^-1^	θ_max_ = 29.1°, θ_min_ = 2.5°
φ and ω scans	*h* = −12→12
Absorption correction: multi-scan (*CrystalClear*; Rigaku, 2008)	*k* = −12→11
*T*_min_ = 0.896, *T*_max_ = 0.985	*l* = −12→13
7468 measured reflections	

### Refinement

**Table:**

Refinement on *F*^2^	Primary atom site location: structure-invariant direct methods
Least-squares matrix: full	Secondary atom site location: difference Fourier map
*R*[*F*^2^ > 2σ(*F*^2^)] = 0.037	Hydrogen site location: inferred from neighbouring sites
*wR*(*F*^2^) = 0.092	H atoms treated by a mixture of independent and constrained refinement
*S* = 1.00	*w* = 1/[σ^2^(*F*_o_^2^) + (0.0451*P*)^2^ + 0.060*P*] where *P* = (*F*_o_^2^ + 2*F*_c_^2^)/3
3601 reflections	(Δ/σ)_max_ = 0.001
191 parameters	Δρ_max_ = 0.42 e Å^−^^3^
0 restraints	Δρ_min_ = −0.22 e Å^−^^3^

### Special details

**Table:**

Geometry. All e.s.d.'s (except the e.s.d. in the dihedral angle between two l.s. planes) are estimated using the full covariance matrix. The cell e.s.d.'s are taken into account individually in the estimation of e.s.d.'s in distances, angles and torsion angles; correlations between e.s.d.'s in cell parameters are only used when they are defined by crystal symmetry. An approximate (isotropic) treatment of cell e.s.d.'s is used for estimating e.s.d.'s involving l.s. planes.
Refinement. Refinement of *F*^2^ against ALL reflections. The weighted *R*-factor *wR* and goodness of fit *S* are based on *F*^2^, conventional *R*-factors *R* are based on *F*, with *F* set to zero for negative *F*^2^. The threshold expression of *F*^2^ > σ(*F*^2^) is used only for calculating *R*-factors(gt) *etc*. and is not relevant to the choice of reflections for refinement. *R*-factors based on *F*^2^ are statistically about twice as large as those based on *F*, and *R*- factors based on ALL data will be even larger.

### Fractional atomic coordinates and isotropic or equivalent isotropic displacement parameters (Å^2^)

**Table:**

	*x*	*y*	*z*	*U*_iso_*/*U*_eq_	
S1	0.29042 (4)	−0.05634 (4)	0.85224 (4)	0.02678 (11)	
N1	0.60522 (14)	0.56001 (14)	0.06550 (13)	0.0283 (3)	
N2	0.29233 (14)	0.23632 (15)	0.29819 (14)	0.0249 (3)	
N3	0.01854 (14)	−0.04570 (15)	0.65642 (14)	0.0303 (3)	
C1	0.51849 (14)	0.31928 (15)	0.34085 (14)	0.0172 (3)	
C2	0.37070 (14)	0.21875 (15)	0.39343 (14)	0.0177 (3)	
C3	0.30635 (14)	0.10607 (15)	0.55414 (14)	0.0181 (3)	
C4	0.38693 (15)	0.09060 (15)	0.65317 (14)	0.0189 (3)	
C5	0.53818 (15)	0.18480 (15)	0.59641 (14)	0.0192 (3)	
C6	0.60130 (14)	0.30197 (15)	0.43875 (14)	0.0174 (2)	
C7	0.75896 (14)	0.41104 (15)	0.36946 (14)	0.0182 (3)	
C8	0.78192 (16)	0.51184 (16)	0.43512 (16)	0.0235 (3)	
H8	0.6965	0.5123	0.5249	0.028*	
C9	0.92966 (17)	0.61171 (16)	0.36943 (17)	0.0272 (3)	
H9	0.9448	0.6805	0.4144	0.033*	
C10	1.05420 (16)	0.61142 (17)	0.23934 (17)	0.0296 (3)	
H10	1.1552	0.6794	0.1954	0.035*	
C11	1.03249 (16)	0.51254 (18)	0.17277 (16)	0.0289 (3)	
H11	1.1184	0.5127	0.0829	0.035*	
C12	0.88490 (15)	0.41272 (16)	0.23729 (15)	0.0225 (3)	
H12	0.8700	0.3453	0.1909	0.027*	
C13	0.57249 (14)	0.45223 (15)	0.18627 (14)	0.0194 (3)	
C14	0.14689 (15)	0.01656 (15)	0.61603 (14)	0.0213 (3)	
C15	0.63082 (17)	0.15707 (17)	0.70024 (16)	0.0271 (3)	
H15A	0.7477	0.1832	0.6354	0.033*	
H15B	0.6043	0.0430	0.7777	0.033*	
H15C	0.6006	0.2270	0.7551	0.033*	
C16	0.2306 (2)	0.0743 (2)	0.95114 (17)	0.0398 (4)	
H16A	0.3277	0.1310	0.9427	0.048*	
H16B	0.1618	0.0086	1.0625	0.048*	
H16C	0.1699	0.1541	0.9019	0.048*	
H2A	0.205 (2)	0.169 (2)	0.330 (2)	0.039 (5)*	
H2B	0.333 (2)	0.301 (2)	0.201 (2)	0.035 (5)*	

### Atomic displacement parameters (Å^2^)

**Table:**

	*U*^11^	*U*^22^	*U*^33^	*U*^12^	*U*^13^	*U*^23^
S1	0.03045 (19)	0.02449 (19)	0.01632 (16)	−0.00576 (13)	−0.00669 (13)	−0.00220 (13)
N1	0.0253 (6)	0.0297 (7)	0.0223 (6)	−0.0050 (5)	−0.0090 (5)	−0.0039 (5)
N2	0.0217 (5)	0.0272 (6)	0.0187 (6)	−0.0074 (5)	−0.0089 (5)	−0.0020 (5)
N3	0.0267 (6)	0.0311 (7)	0.0247 (6)	−0.0071 (5)	−0.0085 (5)	−0.0050 (5)
C1	0.0158 (5)	0.0164 (6)	0.0158 (6)	−0.0001 (4)	−0.0033 (4)	−0.0061 (5)
C2	0.0173 (6)	0.0166 (6)	0.0179 (6)	0.0012 (5)	−0.0058 (5)	−0.0073 (5)
C3	0.0167 (5)	0.0167 (6)	0.0176 (6)	−0.0012 (4)	−0.0043 (5)	−0.0065 (5)
C4	0.0204 (6)	0.0170 (6)	0.0148 (6)	−0.0006 (5)	−0.0050 (5)	−0.0044 (5)
C5	0.0188 (6)	0.0185 (6)	0.0196 (6)	0.0016 (5)	−0.0076 (5)	−0.0076 (5)
C6	0.0152 (5)	0.0169 (6)	0.0190 (6)	0.0014 (4)	−0.0044 (5)	−0.0087 (5)
C7	0.0153 (5)	0.0170 (6)	0.0199 (6)	0.0018 (4)	−0.0075 (5)	−0.0053 (5)
C8	0.0205 (6)	0.0237 (7)	0.0272 (7)	0.0036 (5)	−0.0086 (5)	−0.0126 (6)
C9	0.0278 (7)	0.0197 (7)	0.0382 (8)	0.0016 (5)	−0.0191 (6)	−0.0107 (6)
C10	0.0211 (6)	0.0280 (8)	0.0297 (7)	−0.0061 (5)	−0.0133 (6)	0.0002 (6)
C11	0.0172 (6)	0.0395 (8)	0.0192 (6)	−0.0005 (6)	−0.0036 (5)	−0.0055 (6)
C12	0.0201 (6)	0.0262 (7)	0.0192 (6)	0.0025 (5)	−0.0073 (5)	−0.0084 (5)
C13	0.0156 (5)	0.0222 (7)	0.0205 (6)	−0.0007 (5)	−0.0068 (5)	−0.0094 (5)
C14	0.0232 (6)	0.0193 (7)	0.0172 (6)	−0.0013 (5)	−0.0070 (5)	−0.0046 (5)
C15	0.0262 (7)	0.0279 (8)	0.0243 (7)	−0.0018 (6)	−0.0132 (6)	−0.0054 (6)
C16	0.0481 (10)	0.0412 (10)	0.0213 (7)	0.0046 (7)	−0.0064 (7)	−0.0119 (7)

### Geometric parameters (Å, °)

**Table:**

S1—C4	1.7778 (13)	C7—C12	1.3908 (18)
S1—C16	1.8064 (16)	C7—C8	1.3926 (18)
N1—C13	1.1453 (16)	C8—C9	1.3898 (17)
N2—C2	1.3477 (16)	C8—H8	0.9500
N2—H2A	0.872 (17)	C9—C10	1.378 (2)
N2—H2B	0.849 (17)	C9—H9	0.9500
N3—C14	1.1417 (16)	C10—C11	1.381 (2)
C1—C6	1.4025 (17)	C10—H10	0.9500
C1—C2	1.4181 (15)	C11—C12	1.3904 (17)
C1—C13	1.4380 (16)	C11—H11	0.9500
C2—C3	1.4147 (16)	C12—H12	0.9500
C3—C4	1.3999 (17)	C15—H15A	0.9800
C3—C14	1.4395 (16)	C15—H15B	0.9800
C4—C5	1.4062 (16)	C15—H15C	0.9800
C5—C6	1.4051 (17)	C16—H16A	0.9800
C5—C15	1.5082 (18)	C16—H16B	0.9800
C6—C7	1.4955 (16)	C16—H16C	0.9800
			
C4—S1—C16	100.64 (7)	C7—C8—H8	120.0
C2—N2—H2A	120.8 (11)	C10—C9—C8	120.28 (13)
C2—N2—H2B	122.4 (11)	C10—C9—H9	119.9
H2A—N2—H2B	116.0 (15)	C8—C9—H9	119.9
C6—C1—C2	122.37 (11)	C9—C10—C11	120.13 (12)
C6—C1—C13	120.51 (10)	C9—C10—H10	119.9
C2—C1—C13	116.82 (11)	C11—C10—H10	119.9
N2—C2—C3	122.31 (11)	C10—C11—C12	120.00 (13)
N2—C2—C1	121.85 (11)	C10—C11—H11	120.0
C3—C2—C1	115.74 (11)	C12—C11—H11	120.0
C4—C3—C2	122.01 (11)	C11—C12—C7	120.30 (13)
C4—C3—C14	120.70 (11)	C11—C12—H12	119.8
C2—C3—C14	117.08 (11)	C7—C12—H12	119.8
C3—C4—C5	121.35 (11)	N1—C13—C1	175.53 (13)
C3—C4—S1	116.68 (9)	N3—C14—C3	175.06 (14)
C5—C4—S1	121.96 (10)	C5—C15—H15A	109.5
C6—C5—C4	117.56 (11)	C5—C15—H15B	109.5
C6—C5—C15	121.49 (11)	H15A—C15—H15B	109.5
C4—C5—C15	120.93 (11)	C5—C15—H15C	109.5
C1—C6—C5	120.81 (10)	H15A—C15—H15C	109.5
C1—C6—C7	117.86 (11)	H15B—C15—H15C	109.5
C5—C6—C7	121.33 (11)	S1—C16—H16A	109.5
C12—C7—C8	119.20 (11)	S1—C16—H16B	109.5
C12—C7—C6	119.83 (11)	H16A—C16—H16B	109.5
C8—C7—C6	120.97 (11)	S1—C16—H16C	109.5
C9—C8—C7	120.08 (13)	H16A—C16—H16C	109.5
C9—C8—H8	120.0	H16B—C16—H16C	109.5
			
C6—C1—C2—N2	−179.89 (13)	C13—C1—C6—C5	172.08 (12)
C13—C1—C2—N2	6.36 (19)	C2—C1—C6—C7	178.04 (12)
C6—C1—C2—C3	3.76 (19)	C13—C1—C6—C7	−8.43 (18)
C13—C1—C2—C3	−169.99 (11)	C4—C5—C6—C1	−2.33 (19)
N2—C2—C3—C4	−178.76 (13)	C15—C5—C6—C1	175.93 (12)
C1—C2—C3—C4	−2.42 (19)	C4—C5—C6—C7	178.20 (12)
N2—C2—C3—C14	−3.88 (19)	C15—C5—C6—C7	−3.54 (19)
C1—C2—C3—C14	172.45 (12)	C1—C6—C7—C12	−57.24 (17)
C2—C3—C4—C5	−1.3 (2)	C5—C6—C7—C12	122.25 (14)
C14—C3—C4—C5	−175.95 (12)	C1—C6—C7—C8	122.61 (14)
C2—C3—C4—S1	179.95 (10)	C5—C6—C7—C8	−57.90 (17)
C14—C3—C4—S1	5.26 (17)	C12—C7—C8—C9	−0.5 (2)
C16—S1—C4—C3	−109.99 (12)	C6—C7—C8—C9	179.67 (13)
C16—S1—C4—C5	71.23 (13)	C7—C8—C9—C10	−0.1 (2)
C3—C4—C5—C6	3.7 (2)	C8—C9—C10—C11	0.4 (2)
S1—C4—C5—C6	−177.62 (10)	C9—C10—C11—C12	−0.2 (2)
C3—C4—C5—C15	−174.62 (13)	C10—C11—C12—C7	−0.4 (2)
S1—C4—C5—C15	4.11 (18)	C8—C7—C12—C11	0.7 (2)
C2—C1—C6—C5	−1.4 (2)	C6—C7—C12—C11	−179.41 (12)

### Hydrogen-bond geometry (Å, °)

**Table:**

*D*—H···*A*	*D*—H	H···*A*	*D*···*A*	*D*—H···*A*
N2—H2B···N1^i^	0.849 (17)	2.305 (17)	3.1360 (17)	166.2 (14)
N2—H2A···N3^ii^	0.872 (17)	2.253 (18)	3.0993 (17)	163.4 (15)

Symmetry codes: (i) −*x*+1, −*y*+1, −*z*; (ii) −*x*, −*y*, −*z*+1.

## References

[bb1] Goel, A. & Singh, F. V. (2005). *Tetrahedron Lett.* **46**, 5585–5587.

[bb2] Macrae, C. F., Edgington, P. R., McCabe, P., Pidcock, E., Shields, G. P., Taylor, R., Towler, M. & van de Streek, J. (2006). *J. Appl. Cryst.* **39**, 453–457.

[bb3] Pratap, R. & Ramb, V. J. (2008). *Tetrahedron Lett.* **49**, 3011–3014.

[bb4] Rigaku (2008). *CrystalClear* Rigaku Corporation, Tokyo, Japan.

[bb5] Sheldrick, G. M. (2008). *Acta Cryst.* A**64**, 112–122. 10.1107/S0108767307043930 18156677

[bb6] Singh, F. V., Parihar, A., Chaurasia, S., Singh, A. B., Singh, S. P., Tamrakar, A. K., Srivastava, A. K. & Goel, A. (2009). *Bioorg. Med. Chem. Lett.* **19**, 2158–2161. 10.1016/j.bmcl.2009.02.118 19303291

[bb7] Singh, F. V., Vatsyayan, R., Royb, U. & Goel, A. (2006). *Bioorg. Med. Chem. Lett.* **16**, 2734–2737. 10.1016/j.bmcl.2006.02.012 16503140

[bb8] Westrip, S. P. (2010). *J. Appl. Cryst.* **43**, 920–925.

